# Forecasting the Requirement for Nonelective Hospital Beds in the National Health Service of the United Kingdom: Model Development Study

**DOI:** 10.2196/21990

**Published:** 2021-09-30

**Authors:** Kanan Shah, Akarsh Sharma, Chris Moulton, Simon Swift, Clifford Mann, Simon Jones

**Affiliations:** 1 NYU Grossman School of Medicine New York, NY United States; 2 Icahn School of Medicine at Mount Sinai New York, NY United States; 3 The Royal Bolton Hospital Bolton United Kingdom; 4 Methods Analytics London United Kingdom; 5 University of Exeter Business School Exeter United Kingdom; 6 Taunton & Somerset NHS Foundation trust Taunton United Kingdom; 7 Division of Healthcare Delivery Science Department of Population Health NYU Grossman School of Medicine New York, NY United States

**Keywords:** bed occupancy, clinical decision-making, forecasting, health care delivery, models, time-series analysis

## Abstract

**Background:**

Over the last decade, increasing numbers of emergency department attendances and an even greater increase in emergency admissions have placed severe strain on the bed capacity of the National Health Service (NHS) of the United Kingdom. The result has been overcrowded emergency departments with patients experiencing long wait times for admission to an appropriate hospital bed. Nevertheless, scheduling issues can still result in significant underutilization of bed capacity. Bed occupancy rates may not correlate well with bed availability. More accurate and reliable long-term prediction of bed requirements will help anticipate the future needs of a hospital’s catchment population, thus resulting in greater efficiencies and better patient care.

**Objective:**

This study aimed to evaluate widely used automated time-series forecasting techniques to predict short-term daily nonelective bed occupancy at all trusts in the NHS. These techniques were used to develop a simple yet accurate national health system–level forecasting framework that can be utilized at a low cost and by health care administrators who do not have statistical modeling expertise.

**Methods:**

Bed occupancy models that accounted for patterns in occupancy were created for each trust in the NHS. Daily nonelective midnight trust occupancy data from April 2011 to March 2017 for 121 NHS trusts were utilized to generate these models. Forecasts were generated using the three most widely used automated forecasting techniques: exponential smoothing; Seasonal Autoregressive Integrated Moving Average; and Trigonometric, Box-Cox transform, autoregressive moving average errors, and Trend and Seasonal components. The NHS Modernisation Agency’s recommended forecasting method prior to 2020 was also replicated.

**Results:**

The accuracy of the models varied on the basis of the season during which occupancy was forecasted. For the summer season, percent root-mean-square error values for each model remained relatively stable across the 6 forecasted weeks. However, only the trend and seasonal components model (median error=2.45% for 6 weeks) outperformed the NHS Modernisation Agency’s recommended method (median error=2.63% for 6 weeks). In contrast, during the winter season, the percent root-mean-square error values increased as we forecasted further into the future. Exponential smoothing generated the most accurate forecasts (median error=4.91% over 4 weeks), but all models outperformed the NHS Modernisation Agency’s recommended method prior to 2020 (median error=8.5% over 4 weeks).

**Conclusions:**

It is possible to create automated models, similar to those recently published by the NHS, which can be used at a hospital level for a large national health care system to predict nonelective bed admissions and thus schedule elective procedures.

## Introduction

### Background and Rationale

**Figure 1:**
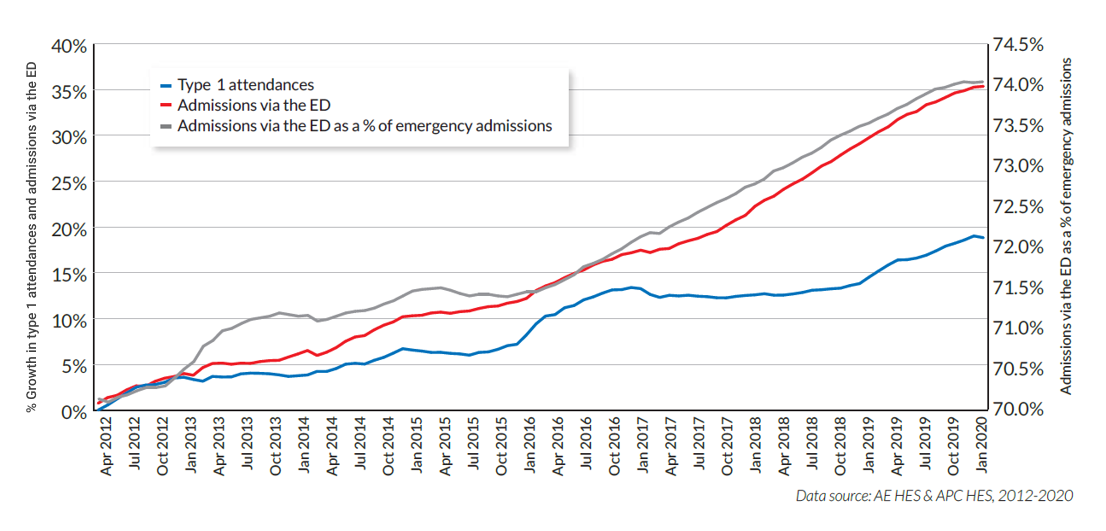
Growth in the admission rate of all nonelective admitted patients. ED: emergency department. Data source: NHS Hospital Episode Statistics for Accident & Emergency and for admitted patient care data, 2012-2020 [10].

Between 2011-2012 and 2019-2020, patient attendances at major (Type 1) emergency departments (EDs) in the National health Service (NHS) of the United Kingdom increased by approximately 20%. There was an even greater increase in the number of patients admitted to hospital from the ED during that time. Such admissions grew by more than one-third and now account for nearly three-fourth of all nonelective admitted patients (Figure 1).

The resulting strain on the bed capacity of the NHS resulted in overcrowding of EDs and long wait times for patients before admission to an inpatient ward. By 2019-2020, over 3.2% of all patients in the ED remained in the ED for more than 12 hours from their time of arrival [1]. Such long delays are known to cause poor patient outcomes, including an increase in all-cause 30-day mortality [2].

For health care systems to meet the increasing needs of the populations they serve, as well as to provide better care, it is imperative to optimize the allocation of existing health care resources, including hospital beds. Health forecasting, a novel area of forecasting, can facilitate this by providing health service providers with the hospital bed occupancy forecasts that will allow them to minimize risks and manage demand [3].

Current models, including NHS-recommended systems, predict hospital bed utilization with a significant degree of error and with marked variability among different hospitals. More accurate and reliable long-term prediction of bed requirements will facilitate the anticipation of a local population’s needs [4] with resulting gains in both efficiency and patient outcomes.

Many studies have attempted to conduct time-series analyses to forecast bed occupancy levels days or weeks in advance [5]. Most of these models use estimates of length of stay [6] or ED admissions [7-9]. We developed models using a more direct time-series–based approach, which utilizes historic admissions data without any identifying patient information, to model nonelective hospital bed requirements. Knowledge of future nonelective bed occupancy allows for proper scheduling of elective procedures to optimize both capacity and resource allocation [6]. In addition, no previous study, to our knowledge, has generated accurate models for an entire national health care system. In this study, we created a modeling framework that is automated and generalizable across the NHS of the United Kingdom. It can be used by administrators and key decision-makers, who have minimal knowledge of statistical techniques, to evaluate and respond more efficiently to clinical demand.

### Objectives

We aimed to (1) test and compare a set of widely used automated time-series forecasting techniques to predict short-term (up to 42 days) nonelective bed occupancy on a daily basis and (2) develop a simple yet accurate system-level forecasting and modeling framework that could be used to predict emergency bed occupancy during different seasonal patterns of admission. A summary of the study rationale and objectives is provided in Textbox 1.

**Summary of the study objectives and rationale.**

**What is already known**

Current statistical models that forecast bed occupancy days or weeks in advance across all trusts in the National Health Service (NHS) of the United Kingdom, including the previously recommended NHS method, predict hospital bed utilization with significant errors and variability among hospitals.

**What this study adds**

We created a modeling framework, following advanced forecasting techniques recommended by the NHS in January 2020, which generates similar forecasts of bed occupancy levels weeks in advance for all trusts in the NHS. In addition, because it is automated and generalizable, this model can be used by administrators and key decision-makers who have a minimal statistical background.

## Methods

### Data

Our data set contained daily nonelective midnight trust occupancy data from April 2011 to March 2017 for 121 NHS trusts located in each region of England. We acknowledge that this is not the same as peak occupancy, which often occurs in the middle of the working day. No personal information on patients or staff was provided. Since these data did not contain any identifying patient information, ethics approval from the institutional review board was not required. In addition, administrative data were utilized, and patients and the public were not involved in our study. We performed all analyses using RStudio (version 1.1.442, RStudio Inc). All generated forecasts accounted for patterns in occupancy or seasonality, resulting from the day of the week being forecasted. One forecast also factored in the day of the year, incidence of public holidays, and historical bed availability.

### Study Design

**Figure 2:**
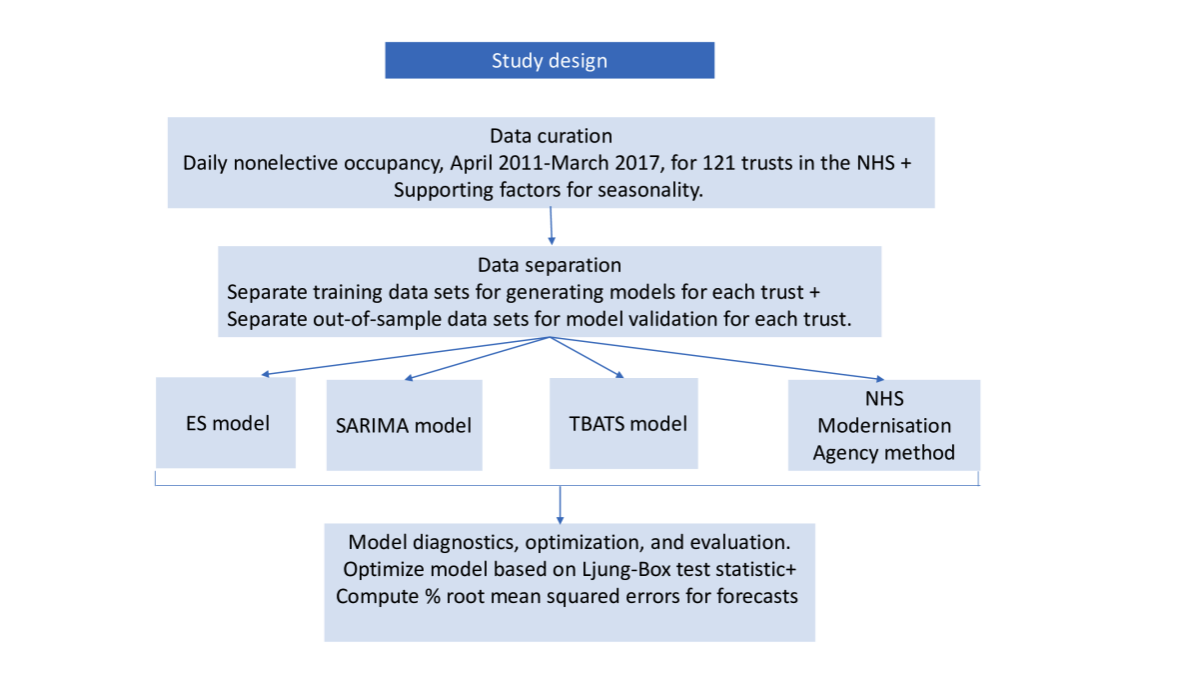
Methodology employed to develop models and generate forecasted nonelective occupancy for each trust in the NHS. ES: exponential smoothing, NHS: National Health Service, SARIMA: Seasonal Autoregressive Integrated Moving Average, and TBATS: Trigonometric, Box-Cox transform, autoregressive moving average errors, and Trend and Seasonal components.

Data preparation and analysis were performed in 4 steps for each forecasting technique employed (Figure 2).

### Data Curation

We extracted daily nonelective occupancy data for 121 trusts in the NHS. To limit our data to general and acute bed occupancy, we excluded admissions in which the consultant’s specialty was related to mental health, learning disabilities, or maternity. The first 10 days’ and last 20 days’ worth of occupancy data for each trust were removed from our data set to account for any edge effects creating inaccuracies in data reporting. The following supporting variables were also included, as they were likely to produce fluctuations or seasonality, in bed occupancy [11]: (1) day of the week, (2) day of the year, (3) public holidays, and (4) historical bed availability.

### Separation of Data

Each trust’s hospital occupancy data were divided into 2 seasonal data sets for summer and winter each, given that hospitals are more burdened during winter months, and this may introduce complications in the forecasting process. Each seasonal data set was further subsetted into a training data set that was used to develop the models and a validation data set that was used to cross-validate the models. The validation data sets for the summer season contained the last 6 weeks of occupancy data for mid-July to mid-August 2016 and those for winter contained the last 6 weeks of occupancy data for February to mid-March 2017. The derivation data sets contained all remaining data from April 2011 to the start date of the validation data sets and were used to develop the models.

### Model Generation and Evaluation

We developed a set of models for each trust by using the three most widely used, automated forecasting techniques: exponential smoothing (ES); Seasonal Autoregressive Integrated Moving Average (SARIMA); and Trigonometric, Box-Cox transform, autoregressive moving average errors, and Trend and Seasonal components (TBATS) (Table 1). Details regarding this model are provided in Multimedia Appendix 1. These models are in line with the NHS Modernisation Agency’s newly released overview of advanced forecasting techniques that can be used to model NHS services [12]. We also replicated the NHS Modernisation Agency’s previously recommended, albeit dated, forecasting method [13].

Therefore, we developed 4 models for each of the 121 trusts in the NHS. A program was developed in R to automate the entire process to minimize repetition and maximize efficiency. Two tests were applied for each forecast: the Ljung-Box test output (Multimedia Appendix 2) to measure for residual patterns of the models’ errors that could be corrected for with additional modeling parameters, and root-mean-square error (RMSE) values from cross-validation. Absolute RMSE values were then converted to percentage errors, representing the average prediction error irrespective of sign (Multimedia Appendix 2). The numerator was the median RMSE of the forecasting method, and the denominator was the total number of general and acute beds in the hospital. The denominator, therefore, was the same for all methods. A comparative analysis of forecast accuracy was performed by comparing forecasted daily nonelective occupancy with actual nonelective occupancy in the out-of-sample data set for each week forecasted.

Forecasts were generated for the summer and winter. Given that summer school holidays in the United Kingdom usually occur from late July until early September and NHS data for England suggest that winter pressures mostly last from early January until the end of March, we forecasted the time periods within those timeframes. Summer forecasts were obtained from July to mid-August 2016 and winter forecasts were obtained from mid-February to mid-March 2017. Forecasts were compared to each other as well as to those derived from the NHS Modernisation Agency’s recommended method.

We did not generate models using Prophet and artificial neural networks, which are 2 additional models recommended in the Modernisation Agency’s overview, because these cannot be automated and applied across multiple trusts [12].

**Table 1:** Descriptive characteristics of automated time-series forecasting techniques used.

Characteristics	Models generated			
	Exponential smoothing	Seasonal Autoregressive Integrated Moving Average	Trend and Seasonal Components	Method recommended by the National Health Service Modernisation Agency
Statistical methods employed	Exponential weighted sum of previous observations	Combines auto-regression and moving average models	State space reconstruction	Forecast is the mean value of the past 6 weeks’ bed occupancy for the day of the week being forecasted
Seasonality taken into account	Weekly	Weekly, yearly, monthly, public holidays, and historical bed availability	Weekly	Weekly
Additional modifications performed	LThe Ljung-Box test provided information on residual patterns of error; this was addressed by creating a model of the residuals of the forecast	Suspected model may be more accurate for trusts that do not approach maximum occupancy (occupancy=<95%); percent occupancy was introduced as a seasonal component	None	None

## Results

**Figure 3:**
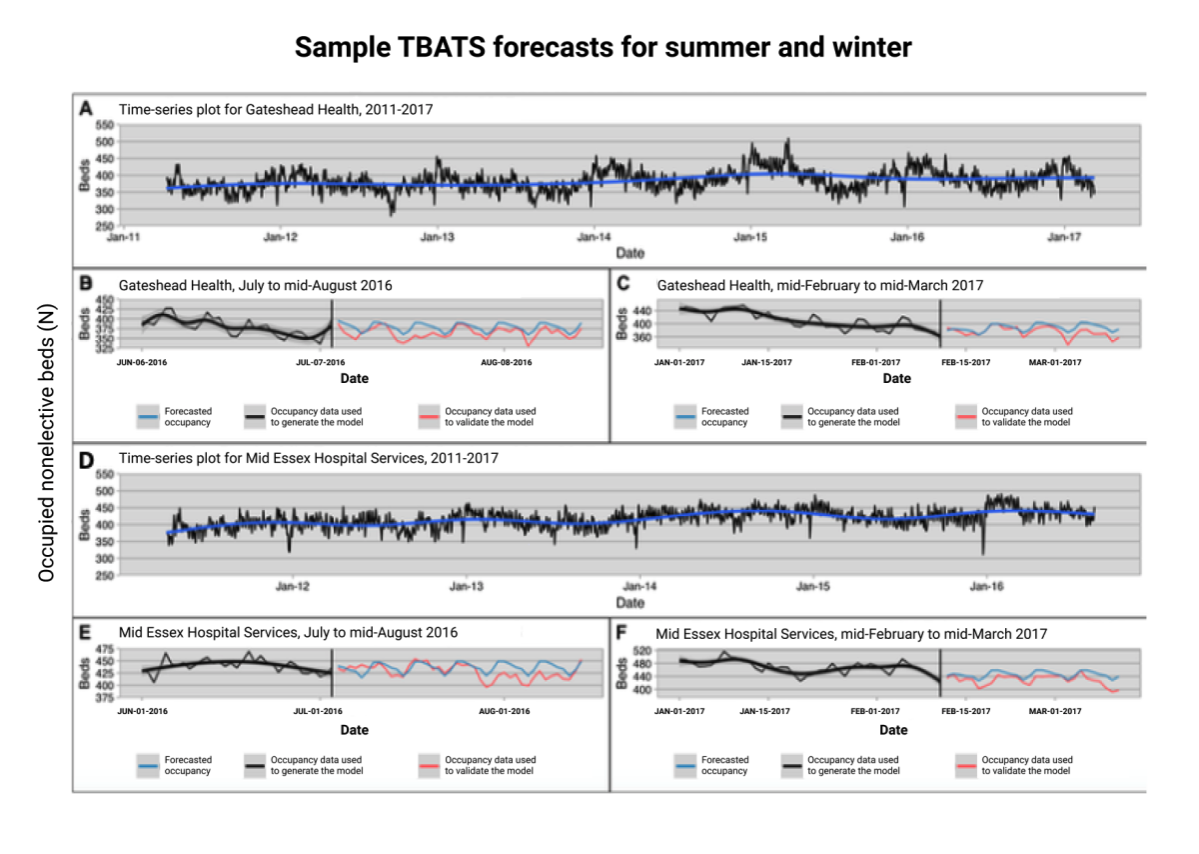
Sample time series and TBATS forecasts generated for summer and winter for two trusts: Gateshead Health (A-C) and Mid Essex Hospital Services (D-F). (A) and (D) show plots of nonelective bed occupancy through the time period in the data set. In (B)-(C) and (E)-(F), the black lines represent the training data sets, the red lines represent the out-of-sample datasets, and the blue lines represent the occupancy forecasted by TBATS. TBATS: Trigonometric, Box-Cox transform, autoregressive moving average errors, and Trend and Seasonal components.

A total of 484 models (n=4 per trust) were automatically developed using our modeling framework (Figure 3). Our Ljung-Box tests validated that autocorrelation is minimal, thus validating our choice of model.

**Figure 4:**
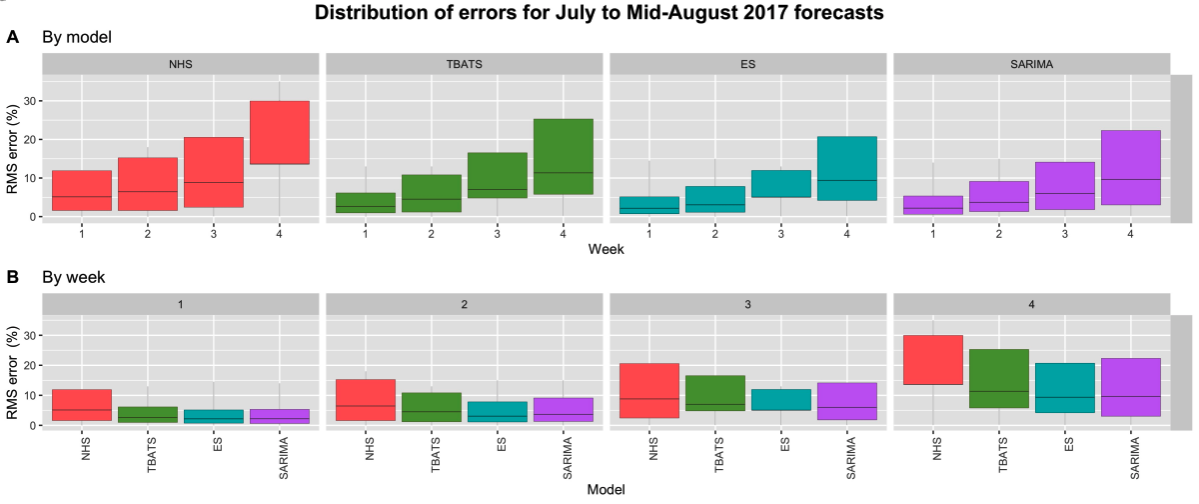
Distribution of error values for summer forecasts displayed by model (A) and by week (B). Outliers have been suppressed in (B) for better visualization of error spread. ES: exponential smoothing, NHS: National Health Service, RMS: root-mean-square, SARIMA: Seasonal Autoregressive Integrated Moving Average, TBATS: Trigonometric, Box-Cox transform, autoregressive moving average errors, and Trend and Seasonal components.

The accuracy of our models varied on the basis of the season during which we forecasted occupancy. Percent RMSE values for each model remained relatively stable across the 6 weeks forecasted in the summer, indicating that the summer period is predictable (Figure 4). In addition, only our TBATS model (median error=2.45% for 6 weeks) outperformed the NHS Modernisation Agency’s recommended method (median error=2.63% for 6 weeks). TBATS yielded a median error of 1.98% for the first forecasted week and 3.01% for the sixth, while the NHS Modernisation Agency’s recommended method yielded a median error of 2.32% for the first forecasted week and 3.17% for the sixth.

**Figure 5:**
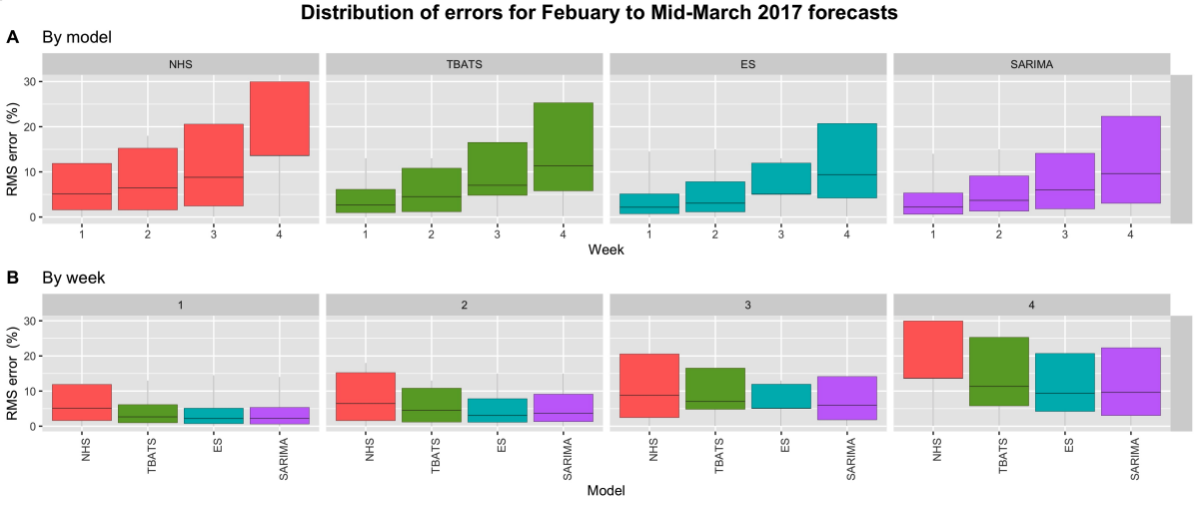
Distribution of error values for winter forecasts displayed by model (A) and by week (B). Outliers have been suppressed in (B) for better visualization of error spread. ES: exponential smoothing, NHS: National Health Service, RMS: root-mean-square, SARIMA: Seasonal Autoregressive Integrated Moving Average, TBATS: Trigonometric, Box-Cox transform, autoregressive moving average errors, and Trend and Seasonal components.

In contrast, percent RMSE values increased as we forecasted further into the future during winter (Figure 5). Therefore, our study suggests that we are only able to generate relatively accurate forecasts 4 weeks into the future during winter. Significant weather events and disease outbreaks may contribute to this unpredictability. However, as current weather forecasting methods are unable to predict significant events accurately beyond 10 days, accounting for weather beyond this is impractical. ES performed the best (median error=4.91% over 4 weeks), but all models outperformed the NHS Modernisation Agency’s recommended method (median error=8.5% over 4 weeks). ES yielded a median error of 2.17% for the first forecasted week and 9.38% for the fourth, while the NHS Modernisation Agency’s recommended method yielded a median error of 5.12% for the first forecasted week and 13.62% for the sixth.

Five or fewer trusts failed to pass the Ljung-Box test of autocorrelation for the TBATS and SARIMA models, which suggested that the models could not be improved much further for accuracy. However, as a large proportion of trusts failed to pass for the ES model (40% for summer forecasts and 42% for winter forecasts), we developed a TBATS model to forecast the residuals of our predictions and incorporated these forecasted residuals into our original model. This modification, however, did not significantly improve forecast accuracy. We also suspected that forecasts may be more accurate for trusts that do not reach their maximum bed availability (less than 95% of total beds are occupied). Therefore, we subsetted trusts on the basis of maximum bed availability and generated separate SARIMA forecasts for each group. This increased each forecast accuracy for the winter but did not affect forecasts much for the summer.

## Discussion

### Principal Findings

Our results show that it is possible to create automated models to predict nonelective bed admissions with a higher degree of accuracy and reliability than the method previously recommended by the NHS Modernisation Agency.

Utilization of the NHS-recommended method has led to the lack of capacity and procedures being canceled at the last minute (Figure 3). Although some of these problems are inevitable in such a large and diffusely managed system, the frequency of such cancellations can be reduced with improved forecasting methods such as the one described in this study.

Other groups that focused on hospitals in England have generated forecasting models for either a single site or a group of trusts, or for specific hospital services such as the emergency department, which is more easily predictable [14]. However, to our knowledge, none of them have utilized the data of the entire NHS to generate more accurate forecasting models [14]. Although individual, site-specific models have the potential to outperform national models, we believe that the majority of hospitals do not have the resources, time, or expertise required to generate their own predictive methodology. Therefore, a more universal and easily implemented—albeit slightly less accurate—modeling framework is preferable. Moreover, our models are automated and require minimal effort for consistent execution.

Even with a simplified approach and appropriate end-user education, several barriers to implementation could limit the use of the developed national forecasting models. In the NHS system, staffing rotas lack the flexibility required to reduce staff at short notice. While an incorrect forecast predicting an increased demand would result in financial losses, an erroneous prediction of a reduction could lead to adverse clinical events. Therefore, caution dictates that users are more likely to respond to forecasts of increased demand rather than those predicting the reverse. Routine forecasting would therefore be likely to increase costs, at least in the initial phase.

If such modeling frameworks are to be incorporated into policy, it is essential to consider whether effective implementation is possible. A recent study of ED escalation plans [15] to support patient care in times of increased demand reported that there can be a significant gap between managerial intentions and actual implementation.

### Limitations

Potential bias may have arisen from inaccurate data collection and reporting at a local level. In addition, our occupancy data were collected as a midnight census rather than during the day, when hospital occupancy peaks. This may limit the applicability of the model. Although our models took into account various temporal models, we did not explicitly consider meteorological conditions such as weather or air pollution—factors that could have an impact on predictive accuracy [16,17].

Another limitation of our study is that we were not able to model demand for outpatient services or elective procedures, which may have a significant impact on the availability of inpatient resources, including health care professionals themselves [18]. Nevertheless, this is an area that clinicians and managers can control to a large extent [13]. In addition, there is a need for caution when predicting the real-world implications of total bed utilization from models in which maximum capacity is approached, as small random effects may have unpredictable consequences.

As these models require automation, we have not utilized the most advanced predictive techniques available. The self-exciting threshold autoregressive model and artificial neural networks would be likely to produce more accurate predications once fully optimized. We explored this model but ultimately rejected it because of the high degree of personalization that the self-exciting threshold autoregressive system required for correct usage. This would thus be impractical for hospital staff with limited statistical knowledge—or the time to acquire it—for effective implementation.

### Conclusions

There is no sign of an imminent reduction in the demand for all hospital services. Therefore, improvements in the efficiency of health care resource utilization are of paramount importance. We believe that, to our knowledge, this is the first study to generate accurate forecasting models for an entire health care system. In addition, our models are automated and require minimal effort to execute consistently and accurately; thus, they are in parallel with the NHS Modernisation Agency’s latest guidelines on advanced forecasting techniques [12]. With increased predictive accuracy of nonelective bed occupancy, more reliable elective procedure schedules can be produced by hospital managers. This increased efficiency should lead to better care for patients, together with a more consistent workflow pattern for health care staff. We believe that a similar methodology can be applied to hospital systems other than the NHS, in other countries including the United States, and we hope to apply these models more widely in the future.

## Appendix

Multimedia Appendix 1 Details regarding the forecasting techniques used in developing the current prediction model.

Multimedia Appendix 2 Supplementary tables.

## Abbreviations

ED: emergency department
ES: exponential smoothing
NHS: National Health Service
RMSE: root-mean-square error
SARIMA: Seasonal Autoregressive Integrated Moving Average
TBATS: Trigonometric, Box-Cox transform, autoregressive moving average errors, and Trend and Seasonal components
