# Supplementary material for: Forecasting the Requirement for Nonelective Hospital Beds in the National Health Service of the United Kingdom: Model Development Study
Source: JMIR Med Inform. 2021 Sep 30;9(9):e21990. doi: 10.2196/21990 (PMC8517824; doi:10.2196/21990)
Supplement: Multimedia Appendix 1 [file medinform_v9i9e21990_app1.docx]

### Appendix

In ES, observations are exponentially weighted according to their distance from the forecasting period. The most recent observations, therefore, have more impact on the forecasted value than past observations. The SARIMA, on the other hand, uses autocorrelations in the time series to generate forecasts. This takes into account autoregression, implying that the prediction depends on previous observations, and the moving average, which takes into account errors in previous data. The SARIMA, unlike ES and TBATS, is also able to account for more than one factor that may be causing seasonal patterns in trust occupancy. In our case, all models took into account the fluctuations that resulted from the day of the week being forecasted, but only the SARIMA took into account the day of the year, the incidence of public holidays and historical bed availability. The TBATS model is a state space modeling framework that incorporates Box-Cox transformations, Fourier representations with time varying coefficients and autoregressive moving average error correction. It allows for the forecasting of complex, seasonal time series. Lastly, in the NHS Modernisation Agency’s recommended method, the mean value of the past six weeks’ occupancy for the day of the week being considered serves as the forecasted value.
