# Supplementary material for: Forecasting the Requirement for Nonelective Hospital Beds in the National Health Service of the United Kingdom: Model Development Study
Source: JMIR Med Inform. 2021 Sep 30;9(9):e21990. doi: 10.2196/21990 (PMC8517824; doi:10.2196/21990)

### Supplementary Tables

Table S2. Ljung-Box Test for Model Diagnostics. Non-zero autocorrelation (p <0.05) suggests that residual information remains in the time series.

###


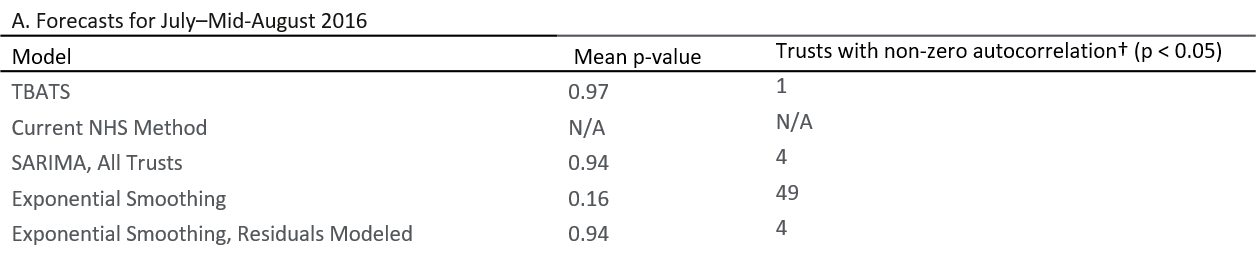

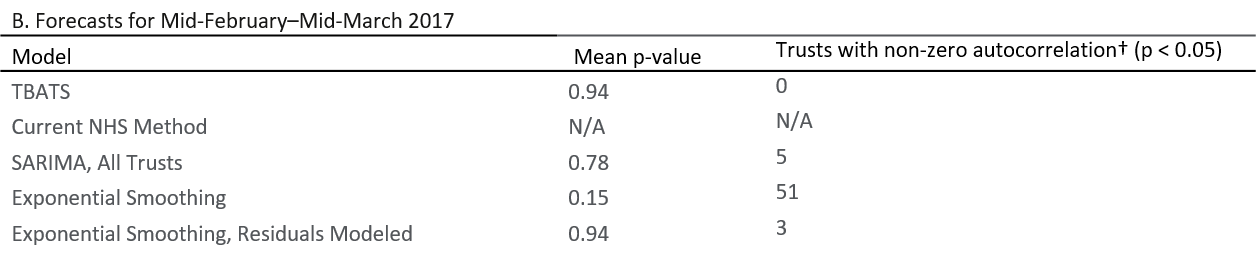


Table S3. Median % RMSE values for Model


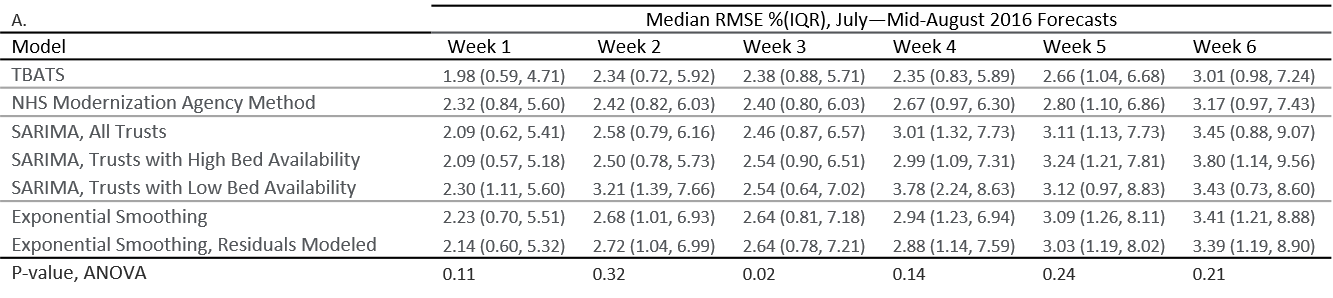

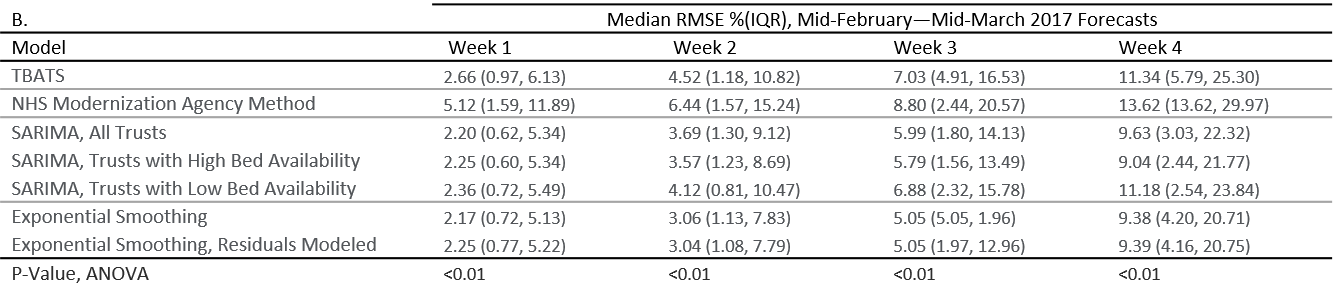

Supplement: Multimedia Appendix 2 [file medinform_v9i9e21990_app2.docx]
